# Slight femoral under-correction *versus* neutral alignment in total knee arthroplasty with preoperative varus knees: a comparative study

**DOI:** 10.1186/s42836-021-00105-4

**Published:** 2022-01-07

**Authors:** Kai Lei, Li-Ming Liu, Peng-Fei Yang, Ran Xiong, De-Jie Fu, Liu Yang, Lin Guo

**Affiliations:** Center for Joint Surgery, Southwest Hospital, Third Military Medical University (Army Medical University), No. 30 Gaotanyan Street, Shapingba District, 400038 Chongqing, China

**Keywords:** Total knee arthroplasty, Knee replacement, Varus knee, Under-correction, Mechanical alignment, Adjusted mechanical alignment, Propensity score matching

## Abstract

**Background:**

This study aimed to compare the short-term clinical results of slight femoral under-correction with neutral alignment in patients with preoperative varus knees who underwent total knee arthroplasty.

**Methods:**

The medical records and imaging data were retrospectively collected from patients who had undergone total knee arthroplasty in our hospital from January 2016 to June 2019. All patients had varus knees preoperatively. Upon 1:1 propensity score matching, 256 patients (256 knees) were chosen and divided into a neutral alignment group (*n*=128) and an under-correction group (*n*=128). The patients in the neutral group were treated with the neutral alignment. In the under-correction group, the femoral mechanical axis had a 2° under-correction. The operative time, tourniquet time and the length of hospital stay in the two groups were recorded. The postoperative hip-knee-ankle angle, frontal femoral component angle and frontal tibial component angle were measured. Patient-reported outcome measures were also compared.

**Results:**

The operative time, tourniquet time and the length of hospital stay in the under-correction group were significantly shorter than the neutral alignment group (*P*<0.05). At the 2-year follow-up, the under-correction group had a larger varus alignment (*P*<0.05) and a larger frontal femoral component angle (*P*<0.05), and the frontal tibial component angles of the two groups were comparable. Compared with the neutral alignment group, the slight femoral under-correction group had significantly better patient-reported outcome measures scores (*P*<0.05).

**Conclusion:**

For varus knees treated with total knee arthroplasty, alignment with a slight femoral under-correction has advantages over the neutral alignment in terms of the shorter operative time and better short-term clinical results.

**Level of evidence:**

III

## Introduction

Total knee arthroplasty (TKA) represents one of the most effective treatments for end-stage knee osteoarthritis. However, patient satisfaction following TKA is less than 80% due to several factors [1, 2]. Among them, the alignment philosophy has aroused particular attention [3–9].

In TKA, restoring neutral mechanical axis alignment has been a longstanding principle, aiming to achieve balanced load distribution and improve the components’ durability and clinical outcomes. However, Bellemans *et al* [10] found that most of the normal knee joints had a constitutional varus deformity, with an average varus hip-knee-ankle (HKA) angle of 1.3°±2.3° (mean ± standard deviation) in healthy adults. About 62% of normal adults had varus knees and 25% of them had larger varus angles (>3°) [10]. Miller *et al* [11] found the static alignment could not be used to predict the dynamic loading of knee joints. The studies on gait showed that the loading of medial and lateral compartments was similar in the involuntary heel touch stage [12, 13]. Therefore, an alignment philosophy named “under-correction” was introduced [14–23], emphasizing an appropriate residual mal-alignment in TKA. Multiple studies showed that the postoperative varus alignment might not influence the clinical outcomes [17, 19, 24, 25]. Slight under-correction (3° to 6° of varus angle) may benefit the clinical outcomes in patients with preoperative varus knees [16, 18].

Currently, under-correction remains a controversial topic because those early studies were based on the relationship between the clinical outcomes and alignment on the basis of the postoperative HKA angle [17, 18, 23]. Until now, few studies have examined whether slight femoral under-correction outperforms the neutral alignment in terms of clinical outcomes.

The purpose of this retrospective study was to compare the slight femoral under-correction with the neutral alignment in patients who underwent TKA with preoperative varus knees. We also compared the outcomes of the two techniques through a short-term follow-up.

## Materials and methods

This study has been approved by the Medical Ethics Committee of the First Hospital Affiliated to Army Medical University, the Chinese People’s Liberation Army (No. KY2020105).

The medical records and imaging data were retrospectively collected from a series of TKAs performed by the same senior surgeon in our hospital from January 2016 to June 2019. The inclusion criteria were (1) knee osteoarthritis; (2) a confirmed preoperative varus knee deformity based on HKA angle; (3) TKAs with personalized three-dimensional reconstruction *as per* preoperative planning [26]; (4) the same primary posterior-stabilized component (Legion® Total Knee System, Smith-Nephew, Inc., Memphis, IN, USA) used. The exclusion criteria were (1) patients without pre- or postoperative full-length weight-bearing radiographs of the lower limbs (*i*.*e*., not meet Paley’s criteria [27]) since it affected the measurement; (2) patients whose follow-up data were not available; (3) the side with better outcomes in bilateral TKAs.

A total of 330 patients (330 knees) were enrolled. Based on the intraoperative alignment targets, the patients were divided into a neutral alignment group (*n*=159) and an under-correction group (*n*=171). A senior surgeon initially followed the mechanical alignment philosophy in this case series and gradually switched to a slight femoral under-correction (2°) in the middle and late stages based on the philosophy of adjusted mechanical alignment [28]. To reduce the influence of selection bias and potential confounding factors, age, sex, side, body mass index (BMI), and preoperative HKA angle were selected to attain a 1:1 propensity-score matching with the caliper set to 0.02. Finally, each group had 128 patients (128 knees).

### TKA in neutral alignment group

Operations were performed under lumbar plexus combined with sciatic nerve block anesthesia and with tourniquet control. We selected Insall’s medial parapatellar approach for TKA. After the tibia was dislocated, we performed a proximal tibial osteotomy perpendicular to the tibia mechanical axis and performed a distal femoral osteotomy perpendicular to the femoral mechanical axis. To ensure the osteotomy accuracy, the procedures were performed on the basis of the personalized preoperative three-dimensional plan [26]. Then we removed the cruciate ligaments, osteophytes, and residual meniscus tissues. To balance the extension gap, we performed a three-step medial release described by Kim* et al* [29] as needed. A femoral rotational osteotomy was conducted with the knee flexed, referring to Whiteside’s line and tibial plateau osteotomy surface. Appropriate femoral size and anteroposterior position were selected to balance flexion gap and extension gap. Once stable balance had been achieved, the prosthesis components were implanted and fixed with bone cement. The tourniquet was released when the joint cavity was rinsed. We did not perform patella replacement.

### TKA in under-correction group

**Fig. 1:**
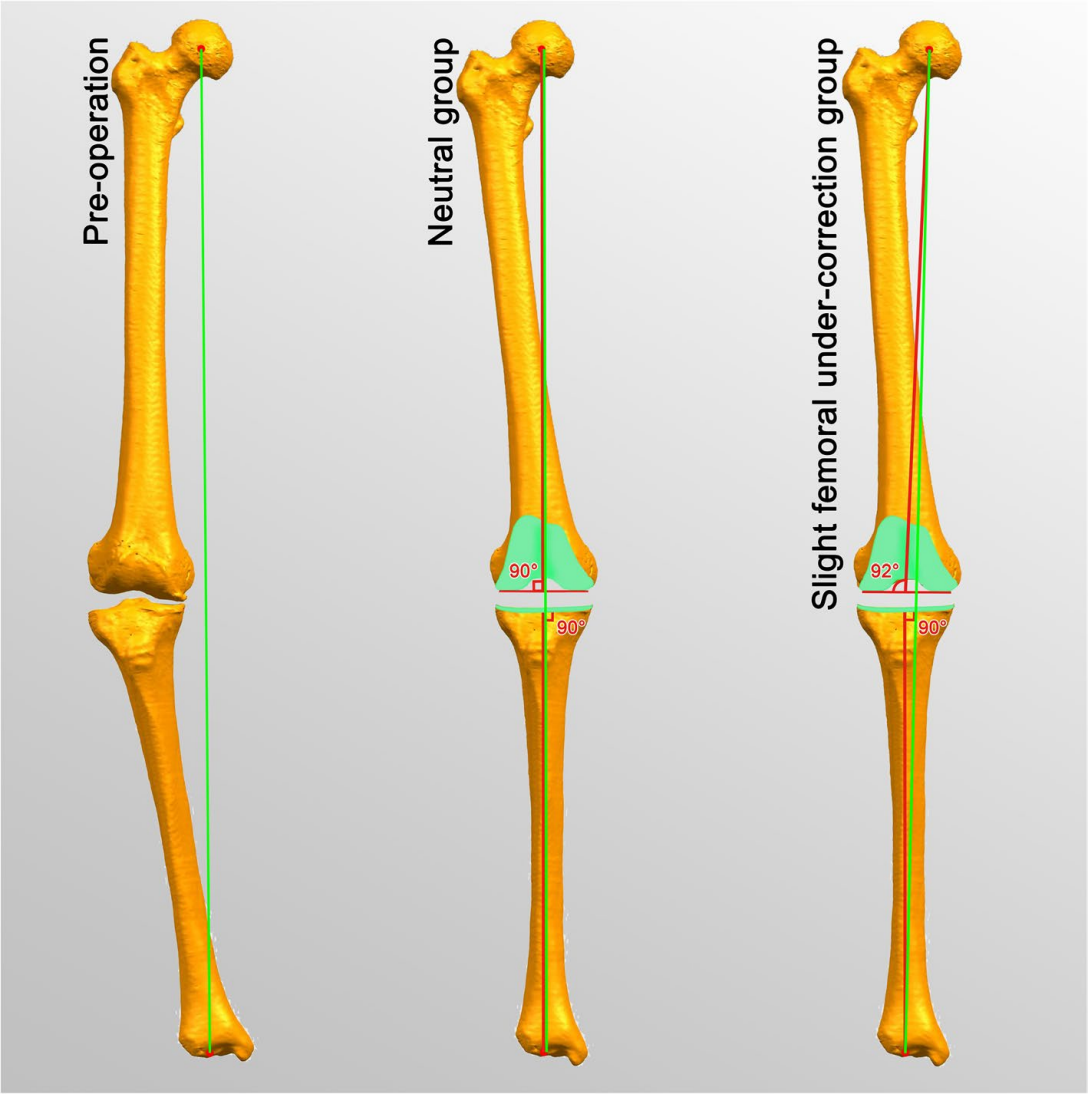
Comparison of alignment targets between two groups

We performed the same surgical procedures except the distal femoral osteotomy. The bone-cutting plane was virtually perpendicular to the femoral mechanical axis with 2° under-correction (Fig. 1).

### Postoperative management

The rehabilitation program was started one day after surgery. Patients were discharged when they met all the following criteria: no obvious swelling, no extension lag, active bending ≥90°, walking distance with assistance ≥200 m, and the visual analogue scale scores ≤ 4 points.

### Outcome evaluation

A full-length weight-bearing radiograph was taken within 1 month before surgery, and the HKA angle was measured [26]. The operative time, tourniquet time, the length of hospital stay and medial soft-tissue release (performed or not) were recorded.

**Fig. 2:**
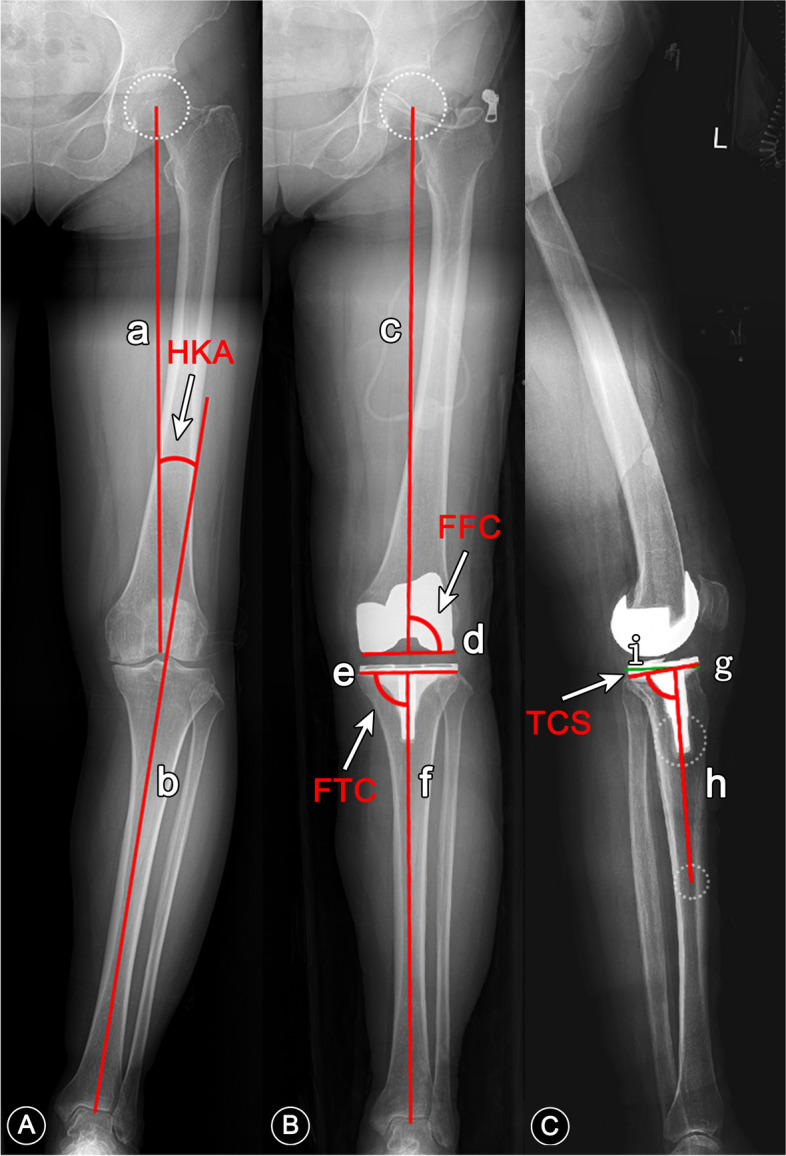
Measurements of HKA angle, FFC, FTC and TCS. HKA: hip-knee-ankle; FFC: frontal femoral component angle; FTC: frontal tibial component angle; TCS: tibial component slope angle; **A** Preoperative HKA angle: the acute angle formed between the femoral mechanical axis (line a) and the tibial mechanical axis (line b). **B** Line c: postoperative femoral mechanical axis; line d: the line across the bottom of the femoral condyles; line e: the line across the bottom of the tibial plateau on the anteroposterior radiograph; line f: the postoperative tibial mechanical axis; FFC: the lateral angle between line c and line d; FTC: the medial angle between line e and line f; postoperative HKA angle: the acute angle between line c and line f. **C** Line g: the line across the bottom of the tibial plateau on the lateral radiograph; line h: the line connecting the center points of the tibial shaft 5 cm and 15 cm below the joint line; line i is perpendicular to line h; TCS: the acute angle between line i and line g

At the 2-year follow-up, a full-length weight-bearing radiograph was taken. The frontal femoral component angle (FFC), frontal tibial component angle (FTC), tibial component slope angle (TCS) was measured three times respectively by two blinded raters, with an interval of more than 15 days. The measurements of HKA angle, FFC, FTC and TCS are shown in Fig. 2. For the HKA angle, the varus angle was negative, and the valgus angle was positive. For tibial component slope angle, retroversion was positive and anteversion was negative. Range of motion of the joints were measured. We collected the data on patient-reported outcome measures (PROMs), including Knee Injury and Osteoarthritis Outcome Score (KOOS), New Knee Society Score (NKSS), and Western Ontario and McMaster Universities Osteoarthritis Index (WOMAC). The WOMAC scores were standardized, ranging from 0 (worst) to 100 (best) based on the guidance proposed by Singh *et al* [30]. These PROMs were obtained preoperatively, 1, 6 month(s) after operation, and once every year thereafter during outpatient follow-ups. Meanwhile, the radiolucent line, aseptic loosening and revision surgery were also recorded and analyzed.

### Statistical analysis

Student *t*-test and chi-square test were performed for continuous and categorical variables, respectively. Intraclass correlation coefficient (ICC) was used to evaluate intra-rater and inter-rater consistency in full-length radiograph measurement. ICC values less than 0.5, between 0.5 and 0.75, between 0.75 and 0.9, and greater than 0.90 were indicative of poor, moderate, good, and excellent reproducibility, respectively [31]. Statistical analysis was performed using SPSS 25.0 (SPSS Inc., Chicago, IL), and a *P*<0.05 was considered statistically significant. In addition, to assess whether statistical significance was clinically significant or not, a minimum clinically important difference (MCID) was introduced [32–35].

## Results

All baseline data were similar between the two groups after propensity score matching (Table 1).

**Table 1:** Comparison of the between-group baseline characteristics before and after PSM

Characteristics	Before PSM (*n* = 330)			After PSM (*n* =256)		
	Neutral group (*n* = 159)	Under-correction group (*n* = 171)	*P*	Neutral group (*n* = 128)	Under-correction group (*n* = 128)	*P*
Gender (Male: Female)	22:137	44:127	**0.007**^**b**^	22:106	19:109	0.609^b^
Side (Left: Right)	79:80	83:88	0.835^b^	64:64	60:68	0.617^b^
Age (year)	63.63±8.45	66.74±8.00	**0.001**^**a**^	64.44±8.72	65.23±7.78	0.441^a^
BMI (kg/m^2^)	25.23±3.17	25.48±3.25	0.472^a^	25.12±3.27	25.53±3.34	0.316^a^
Pre-HKA angle (degree)	-10.89±5.54	-11.72±6.05	0.197^a^	-12.21±5.25	-11.27±6.13	0.190^a^

*PSM* propensity score matching, *BMI* body mass index, *HKA* hip-knee-ankle, For HKA angle, varus was negative and valgus was positive

^a^ stands for student *t*-test

^b^ stands for chi-square test; data are recorded as number or mean ± standard deviation

The intra-rater and inter-rater consistency in full-length radiograph measurement were excellent (ICC>0.9; *P*<0.05). Postoperatively, the under-correction group had a significantly more varus alignment and a larger FFC angle than the neutral group (*P*<0.05). The FTC angles of the two groups were similar (*P*>0.05) (Table 2).

**Table 2:** Comparison of the between-group postoperative alignment and components position at 2-year follow-up

	Neutral group (*n* = 128)	Under-correction group (*n* = 128)	*P*
HKA angle (mean ± SD)	-0.68°±3.35°	-2.56°±2.81°	**<0.001**^**a**^
FFC (mean ± SD)	90.37°±2.33°	91.94°±1.98°	**<0.001**^**a**^
FTC (mean ± SD)	89.72°±2.00°	89.44°±2.10°	0.264^a^
TCS (mean ± SD)	5.16°±3.80°	5.35°±2.59°	0.633^a^
HKA angle <-3°or >3° (*n*)	42	53	0.155^b^
FTC<87°or >93° (*n*)	14	18	0.450^b^

*HKA* hip-knee-ankle, *FFC* frontal femoral component angle, *FTC* frontal tibial component angle, *TCS* tibial component slope angle. For HKA angle, varus was negative and valgus was positive; for TCS, retroversion was positive and anteversion was negative

^a^ stands for student *t*-test

^b^ stands for chi-square test; data are recorded as number or mean ± standard deviation

The operative time, tourniquet time, and the length of hospital stay of the under-correction group were all significantly shorter than those of the neutral group (*P*<0.05). Fewer patients of the under-correction group underwent intraoperative medial soft tissue release (*P*<0.05) (Table 3).

**Table 3:** Comparison of the between-group surgical data

	Neutral group (*n* = 128)	Under-correction group (*n* = 128)	*P*
Operation time (min)	83.74±19.75	73.16±10.95	**<0.001**^**a**^
Tourniquet time (min)	51.67±13.46	42.56±7.28	**<0.001**^**a**^
Length of hospital stay (day)	8.80±1.57	8.27±1.41	**0.004**^**a**^
Soft tissue release (*n*)	59	32	**<0.001**^**b**^

^a^ stands for Student *t*-test

^b^ stands for chi-square test; data are recorded as number or mean ± standard deviation

The preoperative KOOS, NKSS, and WOMAC were similar between the two groups. The under-correction group had significantly better KOOS, NKSS, and WOMAC scores than the neutral group at 2-year follow-up (*P*<0.05) (Table 4). No radiolucent line, aseptic loosening or revision occurred during the whole follow-up (mean: 3.2 years, range: 2-5 years).

**Table 4:** Comparison of the between-group outcomes

		Under-correction group (*n* = 128)	Neutral group (*n* = 128)	Mean difference	*P*
**Flexion range of motion** (degrees)	Pre-op	92.34±17.62	96.09±21.84	-3.75	0.132
	2 years	117.41±6.97	115.89±9.19	1.52	0.139
**KOOS** pain (100 points)	Pre-op	37.26±14.84	37.20±11.71	0.07	0.969
	2 years	90.49±5.57	80.69±10.88	9.8	**<0.001**
Symptoms (100 points)	Pre-op	45.51±15.28	46.01±14.72	-0.5	0.789
	2 years	83.98±7.07	75.47±10.79	8.51	**<0.001**
ADL (100 points)	Pre-op	40.88±13.99	41.96±11.51	-1.08	0.501
	2 years	83.72±7.14	72.85±8.47	**10.87**^a^	**<0.001**
Sport/Rec (100 points)	Pre-op	12.97±15.38	13.40±15.43	-0.43	0.824
	2 years	60.04±15.79	53.36±19.52	6.68	**0.003**
QOL (100 points)	Pre-op	18.28±16.19	18.48±14.16	-0.20	0.918
	2 years	89.18±13.66	78.24±18.94	10.94	**<0.001**
**NKSS** symptom (25 points)	Pre-op	9.55±4.93	9.61±2.64	-0.06	0.912
	2 years	22.06±2.27	19.95±3.76	**2.12**^a^	**<0.001**
Patient satisfaction (40 points)	Pre-op	18.53±6.29	18.61±6.15	-0.08	0.920
	2 years	36.11±2.97	33.53±3.62	**2.58**^a^	**<0.001**
Patient expectations (15 points)	Pre-op	10.83±2.62	10.64±2.55	0.19	0.562
	2 years	11.77±2.43	10.03±2.42	1.73	**<0.001**
Functional activities (100 points)	Pre-op	31.21±15.11	32.16±11.04	-0.95	0.568
	2 years	72.54±6.45	64.97±7.25	**7.57**^a^	**<0.001**
Total score (180 points)	Pre-op	70.13±23.44	71.02±16.49	-0.89	0.725
	2 years	142.48±9.29	128.48±11.90	**14.00**^a^	**<0.001**
**WOMAC** pain (100 points)	Pre-op	44.88±15.23	43.83±12.76	1.05	0.549
	2 years	93.36±4.35	83.98±9.12	9.38	**<0.001**
Stiffness (100 points)	Pre-op	38..67±23.02	36.81±20.56	1.76	0.52
	2 years	82.42±11.89	75.59±18.42	6.84	**<0.001**
Function (100 points)	Pre-op	40.88±13.99	41.96±11.51	-1.08	0.501
	2 years	83.72±7.14	72.85±8.47	**10.87**^a^	**<0.001**
Total score (100 points)	Pre-op	41.53±13.1	41.93±10.36	-0.40	0.787
	2 years	85.62±5.35	75.40±7.48	**10.22**^a^	**<0.001**

*KOOS* Knee Injury and Osteoarthritis Outcome Score, *NKSS* New Knee Society Score, *WOMAC* Western Ontario and McMaster Universities Osteoarthritis Index

^a^ The mean difference was greater than the corresponding minimum clinically important difference; all data were analyzed with Student *t*-test; Data are recorded as mean ± standard deviation

## Discussion

The most important finding of this study was that a slight femoral under-correction (2°) might be associated with shorter operative time and better short-term clinical outcomes than neutral alignment in patients with preoperative varus knees.

Restoring the neutral alignment (known as mechanical alignment) is the prerequisite for a successful TKA. Although the computer-aided techniques such as navigation, patient-specific instrumentation, and robotics have achieved a more accurate neutral alignment, the postoperative patient dissatisfaction is still up to 20% [1]. Unlike the mechanical alignment procedures, the kinematic alignment technique is a “true knee resurfacing” and “pure bone procedure”. The technique aims to reconstruct the anatomy of the pre-arthritic articular surface, thereby improving the clinical outcomes [4, 5, 7, 8]. However, excessively inclined component placement may result in increased load, especially on the tibial component, resulting in quick polyethylene wear, component loosening, and even a revision [36, 37]. Therefore, the concept of adjusted mechanical alignment was introduced [28]. It is a hybrid technique between the mechanical alignment and kinematic alignment procedures, conducting slight under-correction only on the femoral side [28, 38–40]. This technique aims at less collateral releasing and resurfacing the knee within the original soft tissue envelope. As shown in Table 2, postoperative varus alignment in the under-correction group mainly originated from the femoral side. It was consistent with our preoperative planning and the adjusted mechanical alignment philosophy.

Slight femoral under-correction with certain amount of varus deformity preserved reduces the need for medial soft tissue release, resulting in shorter operative time in balancing the soft tissue. Minimal intraoperative soft tissue trauma and short tourniquet time are associated with short hospital stay.

Although slight femoral under-correction procedure has achieved higher KOOS, NKSS, and WOMAC scores after 2 years, statistical significance could not represent perceptive clinical significance. Blevins *et al* [33] found that MCID of KOOS were 10.3 for pain, 12.0 for symptoms, 10.0 for ADL, 15.8 for Sport/Rec, and 13.2 for QOL. MCID for NKSS scores was 1.9 in symptom, 2.2 in satisfaction, and 4.1 in functional activities [34]. Parratte *et al* [35] believed that the differences smaller than 10 points in total NKSS scores were unlikely to be clinically relevant. Clement *et al* [32] demonstrated that the MCID of WOMAC was 11 for pain, 8 for stiffness, 9 for function, and 10 for the total score. Clinical differences were indeed found between the two groups in some sub-measures (mean difference was greater than the corresponding MCID), as shown in Table 4.

There are possible reasons for the slight femoral under-correction outdoing the neutral alignment procedure in terms of short-term PROMs. The majority of the normal population have a slight constitutional varus knee alignment with an average angle of 1.3° [10]. Many patients have a long history of constitutional varus deformity before varus knee osteoarthritis [20]. The mechanical alignment technique aims to create a “systematic approach” rather than restoring the patient’s normal knee structure because it changes the original soft tissue envelope of the knee joint [10, 23, 41]. Surgical correction of the lower limb alignment to the neutral position, specifically, in combination with medial release, may create a relatively valgus status compared to the constitutional varus deformity. The patients may feel unnatural and uncomfortable for a short period of time immediately after TKA. Besides, varus knees need more soft tissue release to attain neutral alignment, while the medial soft tissue release impairs the knee stability and affects postoperative function and rehabilitation [42–44]. Preserving certain level of varus deformity in preoperative varus knees may accomplish a more natural and comfortable status, thereby, improving patient satisfaction [7, 24, 45–48].

Parratte *et al* [49, 50] and Bonne *et al* [49, 50] found that varus alignment less than 3° did not improve the survival of the component after 15 years. Magnussen *et al* [3, 24] and Berend *et al* [3, 24] believed that the varus position of tibial components should be avoided, especially a varus angle exceeding 3°. Therefore, slight femoral under-correction may not significantly decrease the longer-term survival of the components based on Table 2.

This study has some limitations. First, the retrospective design and single-center study might produce selection biases even though propensity score matching was performed. Second, the surgeons’ skill and experience might influence the results because they improve with time. Third, our short follow-up period may not reflect the actual survival rate, and future studies based on mid- to long-term follow-ups should be performed to ascertain the component survival better.

## Conclusion

For varus knees treated with TKA, alignment with a slight femoral under-correction has advantages over the neutral alignment in terms of the shorter operative time and better short-term clinical results.

## Data Availability

All data and materials are available on reasonable request.

## Abbreviations

TKA: Total knee arthroplasty
HKA: Hip-knee-ankle
BMI: Body mass index
FFC: Frontal femoral component angle
FTC: Frontal tibial component angle
TCS: Tibial component slope angle
PROMs: Patient-reported outcome measures
KOOS: Knee Injury and Osteoarthritis Outcome Score
NKSS: New Knee Society Score
WOMAC: Western Ontario and McMaster Universities Osteoarthritis Index
ICC: Intraclass correlation coefficient
MCID: Minimum clinically important difference
